# Age-stratified reporting of immune checkpoint inhibitor-associated myocarditis: disproportionality is amplified in older adults, with implications for geriatric oncology

**DOI:** 10.3389/fcvm.2026.1948161

**Published:** 2026-10-13

**Authors:** Murat Pehlivan, Aybüke Uyar, Okan Turhan, Özge Özgün, Oğuz Kayabaşı, Arzu Okyar Baş, Cafer Balcı, Burcu Balam Doğu, Meltem Gülhan Halil, Mustafa Cankurtaran, Mert Eşme

**Affiliations:** Department of Internal Medicine, Division of Geriatrics, Hacettepe University Faculty of Medicine, Ankara, Türkiye

**Keywords:** cardio-oncology, cardiotoxicity, FAERS, geriatric oncology, immune checkpoint inhibitors, myocarditis, older adults, pharmacovigilance

## Abstract

**Background:**

Cardiovascular toxicity is the immune-related adverse event with the highest reported fatality proportion among immune checkpoint inhibitor (ICI) complications; myocarditis mortality reaches 50%. ICI use has expanded into older populations underrepresented in pivotal trials, but cardiotoxicity reporting in these patients has not been quantified.

**Methods:**

ICI-associated cardiac adverse event records submitted to the FDA Adverse Event Reporting System (FAERS) between January 2011 and March 2026 were retrieved via openFDA. Six ICI agents (pembrolizumab, nivolumab, ipilimumab, atezolizumab, durvalumab, cemiplimab) were crossed with 18 MedDRA Preferred Terms across six cardiac event categories; myocarditis terms were combined at the unique-report level. Reporting odds ratios (RORs) for myocarditis were calculated against the full FAERS background and stratified by age (<65 vs. ≥65 years). RORs reflect reporting disproportionality and do not represent true clinical incidence.

**Results:**

We identified 4,107 unique ICI-associated myocarditis reports and 18,261 cardiac AE records. Among 3,996 age-recorded myocarditis reports, 63.4% occurred in patients aged ≥65 and 28.3% in ≥75 — far exceeding pivotal-trial participation of these age groups. The pooled myocarditis ROR was 34.21 (95% CI 32.95–35.51); agent-specific RORs ranged from 18.73 (atezolizumab) to 41.99 (cemiplimab). Reporting odds for myocarditis were higher in patients aged ≥65 than in those aged <65 for every agent examined; among the five agents with adequate numbers in both strata the amplification ranged from 1.6- to 3.1-fold. Cemiplimab, whose 51 myocarditis reports include 30 with a recorded age and only 3 in the <65 comparison stratum, returned the largest values on every age-related metric; on denominators of that size these are not stable estimates and are reported in the main text rather than here.

**Conclusions:**

ICI-associated myocarditis reporting is disproportionately concentrated in older adults, and the disproportionality signal is amplified in this group across all six agents. The class-wide age effect, rather than the estimate for any single agent, is the principal finding. Older adults appear disadvantaged on two fronts: under-representation in the pivotal trials that established ICI safety profiles, and an age-amplified pharmacovigilance signal. Integrating pre-ICI cardiac evaluation into geriatric oncology practice is a priority as ICI use expands further into older populations.

## 1 Introduction

Immune checkpoint inhibitors (ICIs) are monoclonal antibodies targeting cytotoxic T-lymphocyte-associated antigen 4 (CTLA-4), programmed cell death protein 1 (PD-1), or its ligand (PD-L1). Since the 2011 approval of ipilimumab for advanced melanoma (1, 2), ICIs have become standard of care for multiple solid tumors. The class has since expanded to over 100 approved indications across more than a dozen tumor types, and approximately 44% of patients with cancer in the United States are now eligible for an ICI-containing regimen (3).

Cancer is, increasingly, a disease of old age. Approximately 58% of cancer diagnoses in the United States occur in adults aged ≥65, and cancer is the leading cause of death in adults aged 60–79 years (4). For several first-line ICI indications (non-small-cell lung cancer, urothelial carcinoma, head and neck cancer, hepatocellular carcinoma, and cutaneous squamous cell carcinoma), the median age at diagnosis exceeds 70 (5). The population now receiving ICIs in routine practice therefore differs substantially from the cohort that established each agent's safety profile in registration trials, where adults aged ≥75 typically constituted fewer than 15% of participants (6).

Among the immune-related adverse events that accompany this class, cardiovascular toxicity is associated with the highest reported fatality proportion (7, 8). ICI-associated myocarditis occurs in only 0.09%–2.4% of treated patients in clinical-trial populations (9, 10), but historical mortality rates have approached 50% (9, 11). ICIs may also precipitate pericarditis, arrhythmias, conduction disturbances, accelerated atherosclerosis, and acute coronary events (12, 13). The 2022 European Society of Cardiology (ESC) cardio-oncology guideline accordingly recommends baseline cardiac evaluation before ICI initiation and structured surveillance during therapy (14).

Older adults face several structural disadvantages in this clinical landscape. The aging cardiovascular system shows reduced myocardial contractile reserve, increased fibrosis and stiffening of the conduction system, and impaired vasoreactivity (15). This substrate lowers tolerance to any acute insult, including immune-mediated injury. Aging is also accompanied by declining thymic output, expanded clonal CD8+ T-cell populations, and loss of peripheral immune tolerance (16, 17), changes that may modify the risk of T-cell-mediated cardiotoxicity itself. Multimorbidity, polypharmacy, and frailty further complicate the picture: cardiovascular symptoms attributed to chronic comorbidity may delay recognition of acute drug-related injury, and presentation may be atypical, with non-specific fatigue, exertional intolerance, or vague chest discomfort rather than the textbook acute coronary picture more common in younger patients (18). Despite these factors, ICI cardiotoxicity has been studied predominantly in unselected populations, and its specific magnitude and mechanism in older patients have not been characterized.

Several recent FAERS-based pharmacovigilance analyses have documented the rise in ICI myocarditis reporting and confirmed a strong disproportionality signal across the class (19–23). Reeves and colleagues (20) carried out the most recent and largest such analysis, identifying 1,326 ICI myocarditis cases (after exclusion of duplicates and reports without recorded age) between 2012 and the first quarter of 2023, with median patient age 69 years and a 37% case-fatality rate that has gradually improved with growing clinical experience. Their pooled reporting odds ratio (ROR) for myocarditis across the ICI class was 30.1 (95% CI 28.4–32.0). A real-world Spanish study by Toribio-García and colleagues (24) examined 195 patients aged ≥70 receiving ICIs and reported 1.5% cardiotoxicity incidence in this population, but did not characterize disproportionality or compare with younger comparators.

No previous analysis has stratified ICI-associated cardiotoxicity reporting by patient age in a way that informs geriatric clinical practice. A further open question is whether the disproportionality signal itself is age-dependent: that is, whether older patients are overrepresented in myocarditis reports beyond what differential exposure would predict. We therefore conducted a geriatric oncology–grounded analysis with two aims: (i) to map the temporal trajectory of ICI-associated cardiac adverse event reporting in FAERS across the full cardiac event spectrum; and (ii) to characterize the age structure of affected patients and the age-stratified disproportionality signal across all six commercial ICI agents. Together, these analyses quantify the reporting burden of ICI cardiotoxicity borne by older adults and its implications for geriatric oncology practice.

## 2 Materials and methods

### 2.1 Data source: the FDA adverse event reporting system

Adverse event records were retrieved through the openFDA application programming interface (https://api.fda.gov), which exposes the publicly releasable FAERS dataset in JSON format (25); the full query specification is provided in Supplementary Methods S1. The capture window spanned 1 January 2011 (corresponding to the first FDA approval of an ICI agent, ipilimumab) through 31 March 2026. Six ICI agents were searched by generic name: pembrolizumab, nivolumab, ipilimumab, atezolizumab, durvalumab, and cemiplimab. Eighteen MedDRA Preferred Terms were searched in the patient.reaction.reactionmeddrapt field, grouped clinically into six categories: myocarditis (myocarditis, autoimmune myocarditis, immune-mediated myocarditis); pericardial disease (pericarditis, pericardial effusion); heart failure/cardiotoxicity (cardiac failure, cardiac failure acute, cardiac failure congestive, cardiotoxicity); arrhythmia (atrial fibrillation, ventricular tachycardia, ventricular fibrillation, atrioventricular block complete, arrhythmia); cardiac arrest; and myocardial infarction (myocardial infarction, acute myocardial infarction). Takotsubo cardiomyopathy was searched but excluded from primary analyses because of low case counts.

Two retrieval strategies were used. For the descriptive cross-category overview (Table 1, Figure 1), drug × Preferred Term records were retrieved separately; counts therefore represent drug–reaction records, and a single report contributing two Preferred Terms within the same category may appear twice. For myocarditis-specific analyses (Sections 3.2–3.4, Tables 2, 3, Figures 2, 3, 4), the pooled unique-case total (4,107) was obtained by an OR-combined query of the three myocarditis Preferred Terms at the whole-cohort level, de-duplicating reports containing more than one of these terms. Per-agent unique counts are reports carrying the primary “myocarditis” Preferred Term, retrieved at the report rather than the record level; the two narrower terms (autoimmune myocarditis, immune-mediated myocarditis) are retained in the record-level analyses of Table 1 and Figure 1 but are not added to the per-agent unique counts, which are therefore conservative. For each retrieval, four data layers were extracted: total report count; age distribution at event onset (patient.patientonsetage); annual reporting volume (from receivedate); and seriousness/death status (seriousnessdeath). For the disproportionality analysis, the OR-combined myocarditis query was applied without drug filter to obtain total myocarditis reports across all FAERS submissions (yielding the pooled 4,107 unique-case count), and without reaction filter to obtain total reports per drug. The full FAERS background for the study period contained approximately 19.4 million reports.

**Table 1:** ICI-associated cardiac adverse event drug–reaction records by agent and reaction category (FAERS, January 2011–March 2026).

ICI agent	All cardiac AE records	Myocarditis (unique)	Pericardial	HF/cardiotox.	Arrhythmia	Cardiac arrest	MI
Pembrolizumab	6,632	1,897	617	1,477	948	279	726
Nivolumab	5,904	1,547	686	1,123	1,086	274	663
Ipilimumab	2,600	785	233	398	496	143	261
Atezolizumab	1,995	457	221	491	320	114	334
Durvalumab	1,017	316	100	194	144	47	135
Cemiplimab	113	51	8	17	16	7	9
TOTAL	18,261	4,107	1,865	3,700	3,010	864	2,128

Counts in non-myocarditis columns and the ‘All cardiac AE records' column represent drug–reaction MedDRA Preferred Term records: a single report contributing more than one Preferred Term within a category may be counted more than once. The Myocarditis column reflects unique reports carrying the primary “myocarditis” Preferred Term; the pooled total (4,107) is the de-duplicated OR-combined count across the three myocarditis Preferred Terms. Because the ‘All cardiac AE records' column counts records across all eighteen Preferred Terms, including the two narrower myocarditis terms, the six category columns do not sum to the row total; the shortfall equals the records coded with a narrower myocarditis term (1,641 across all agents). Pericardial = pericarditis + pericardial effusion. HF/cardiotox. = cardiac failure variants + cardiotoxicity. Arrhythmia includes atrial fibrillation, ventricular tachycardia/fibrillation, atrioventricular block, and arrhythmia (unspecified). MI = myocardial infarction (acute and unspecified).

**Figure 1:**
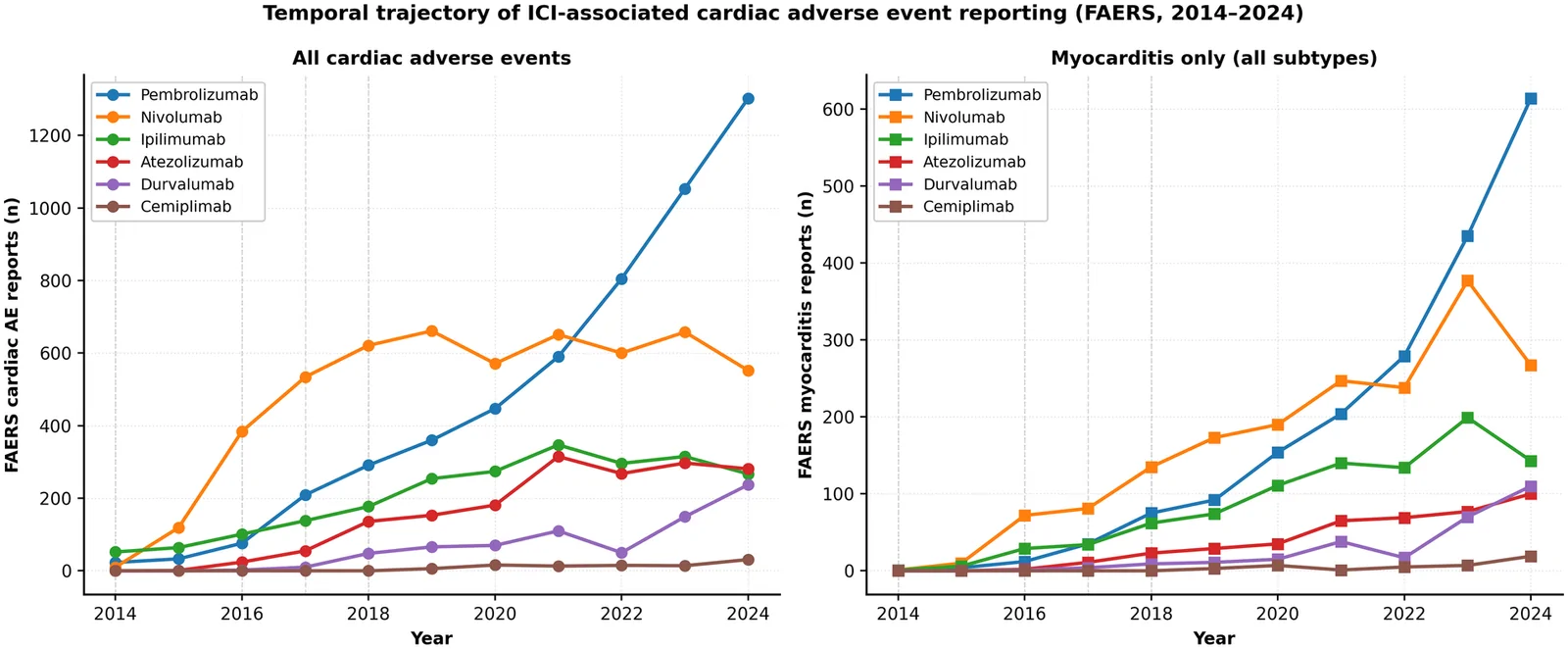
Temporal trajectory of ICI-associated cardiac adverse event reporting (FAERS, 2014–2024). Left panel: annual record counts for all cardiac AE categories combined, by ICI agent. Right panel: annual record counts for myocarditis only (all subtypes combined). Vertical dashed lines mark FDA approval years for each agent. No agent shows a plateau in reporting through 2024.

**Table 2:** Age stratification of ICI-associated unique myocarditis reports (FAERS, January 2011 – march 2026).

ICI agent	Reports with known age	<65 yrs (n, %)	65–74 yrs (n, %)	≥75 yrs (n, %)	≥65 yrs total (%)
Pembrolizumab	1,588	651 (41.0%)	502 (31.6%)	435 (27.4%)	59.0%
Nivolumab	1,245	399 (32.0%)	461 (37.0%)	385 (30.9%)	68.0%
Ipilimumab	626	213 (34.0%)	248 (39.6%)	165 (26.4%)	66.0%
Atezolizumab	314	137 (43.6%)	113 (36.0%)	64 (20.4%)	56.4%
Durvalumab	193	58 (30.1%)	71 (36.8%)	64 (33.2%)	69.9%
Cemiplimab	30	3 (10.0%)	11 (36.7%)	16 (53.3%)	90.0%
POOLED (sum of per-agent counts)	3,996	1,461 (36.6%)	1,406 (35.2%)	1,129 (28.3%)	63.4%

Unique report counts. The pooled total (4,107) was obtained by an OR-combined query of the three myocarditis Preferred Terms (myocarditis, autoimmune myocarditis, immune-mediated myocarditis) at the whole-cohort level; per-agent rows count reports carrying the primary “myocarditis” Preferred Term. The POOLED row of this table reports the arithmetic sum of the per-agent counts (3,996); because combination-therapy reports contribute to each component drug, this sum exceeds the pooled OR-combined unique count. Per-agent age completeness ranges from 59% (cemiplimab) to 84% (pembrolizumab); overall age availability across the 18,261 ICI cardiac adverse event records is 79.5% (14,516 records with a recorded age value; Supplementary Table S8). See Supplementary Table S1 for sensitivity analysis. Pooled fatality rate for ICI myocarditis: 32.7% (1,343/4,107).

**Table 3:** Disproportionality of ICI-associated myocarditis: overall and age-stratified reporting odds ratios (FAERS, 2011–march 2026).

ICI agent	Overall ROR (95% CI)	ROR ≥65 yrs (95% CI)	ROR <65 yrs (95% CI)	Ratio (≥65/<65)
Pembrolizumab	33.34 (31.74–35.02)	42.07 (38.96–45.42)	19.46 (17.94–21.11)	2.16
Nivolumab	27.33 (25.90–28.83)	38.32 (35.40–41.48)	12.39 (11.19–13.72)	3.09
Ipilimumab	29.36 (27.30–31.58)	38.96 (35.09–43.26)	13.58 (11.84–15.58)	2.87
Atezolizumab	18.73 (17.05–20.57)	15.32 (13.16–17.85)	9.72 (8.20–11.52)	1.58
Durvalumab	26.85 (23.99–30.05)	28.87 (24.25–34.37)	16.08 (12.39–20.87)	1.80
Cemiplimab	41.99 (31.76–55.50)	69.71 (47.20–102.96)	13.24 (4.24–41.38)	5.27
ALL ICI POOLED	34.21 (32.95–35.51)	—	—	—

Reporting odds ratios calculated using the standard 2 × 2 disproportionality table against the full FAERS background (≈19.4 million reports) for the 2011–March 2026 window. The numerator a (drug + myocarditis cases) was obtained through an OR-combined query of the three myocarditis Preferred Terms at the unique-report level. Age-stratified analyses use the corresponding age-restricted background totals. The Ratio column expresses the fold-amplification of the ROR in the ≥65-year stratum relative to the <65-year stratum. All ROR point estimates exceed the conventional disproportionality signal threshold of 2 with the lower 95% CI bound above 1. Comparison reference: Reeves et al. (20) reported a pooled ROR of 30.1 (95% CI 28.4–32.0) for the 2012–Q1 2023 window without age stratification.

**Figure 2:**
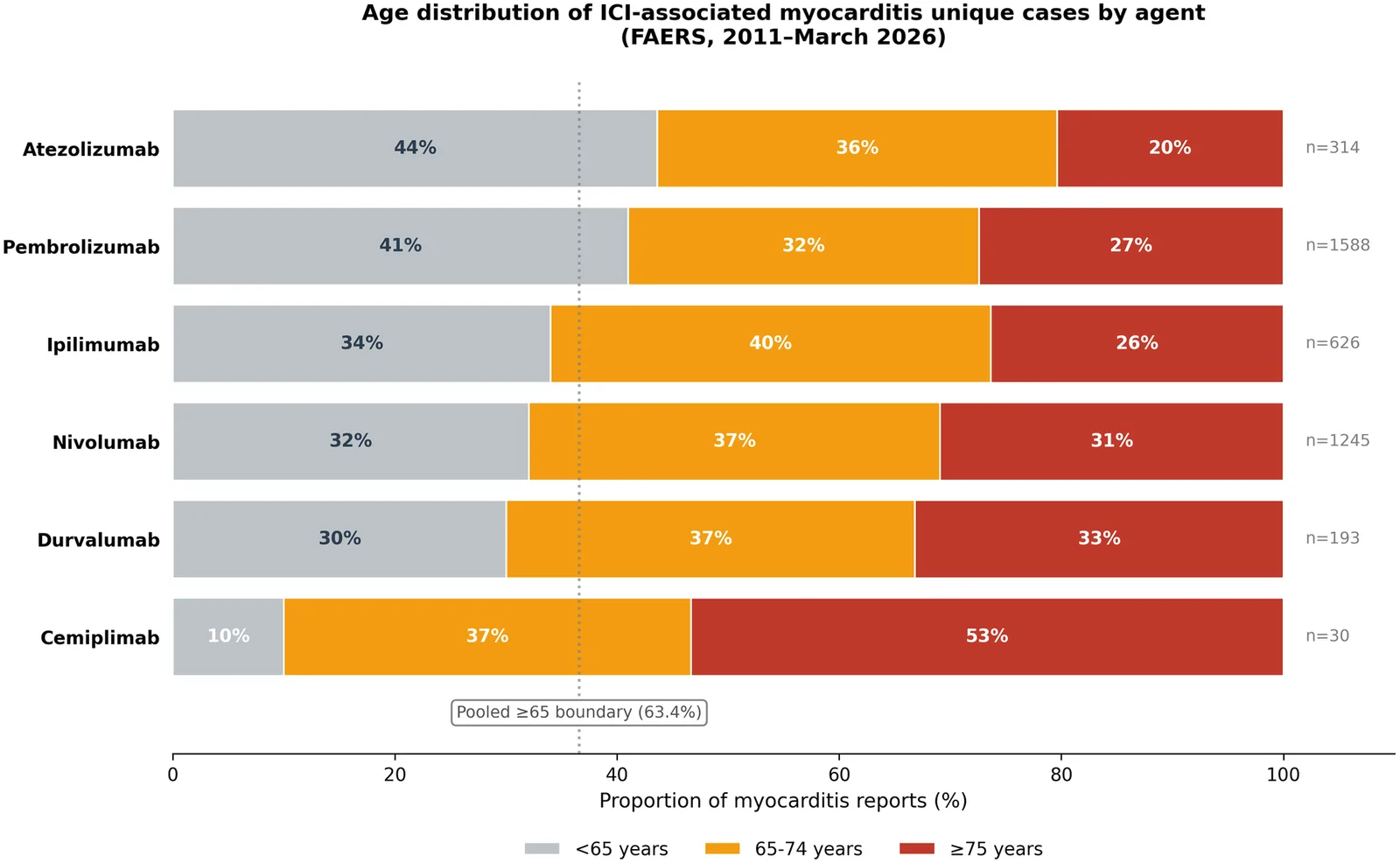
Age distribution of ICI-associated myocarditis unique cases by agent (FAERS, 2011–2026). Stacked horizontal bars show the proportion of unique myocarditis reports in three age strata: <65 years (gray), 65–74 years (orange), and ≥75 years (red). Total unique myocarditis case count with recorded age **(n)** shown to the right of each bar; unique cases (not drug-reaction records) identified through an OR-combined MedDRA Preferred Term query. Cemiplimab shows the most pronounced age skew in the reporting pattern (90.0% of reports in patients aged ≥65 and 53.3% in those aged ≥75) but contributes only 30 age-recorded reports, the smallest denominator of the six agents, so its proportions are correspondingly imprecise..

**Figure 3:**
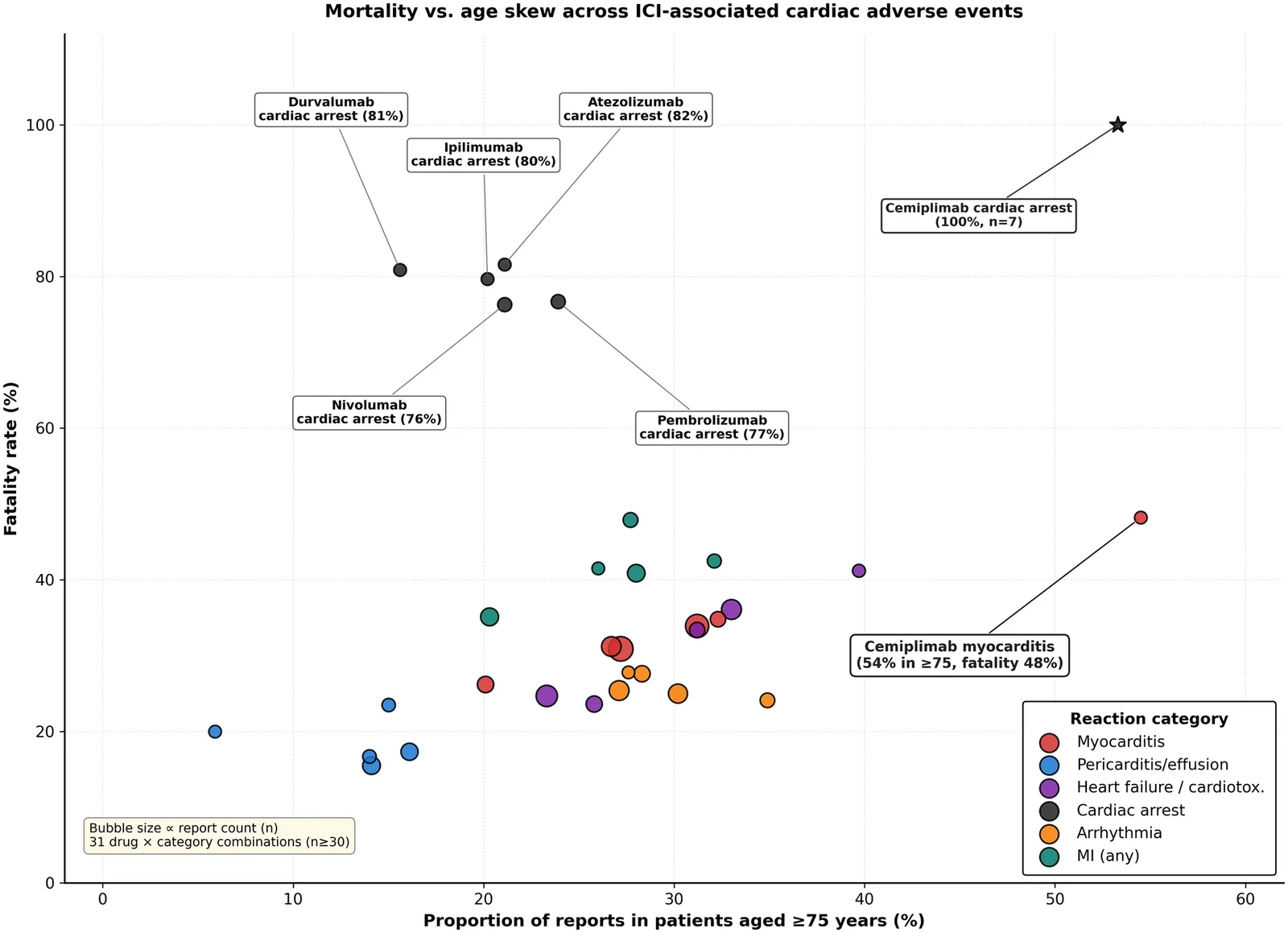
Mortality versus age skew across ICI-associated cardiac adverse event categories (FAERS, 2011–2026). Each bubble shows one ICI agent × category combination with ≥30 records. *X*-axis: proportion of records in patients aged ≥75 years, computed over records with an age recorded in years (Supplementary Table S8). *Y*-axis: proportion of reports with fatal outcome (referred to as fatality rate in axis label). Bubble size proportional to total drug-reaction record count (not unique cases; a single report contributing multiple Preferred Terms may be counted more than once). Categories color-coded as in legend. Cardiac arrest events cluster in the upper portion (76%–82% fatality) across the five agents plotted; cemiplimab cardiac arrest (7 of 7 fatal) falls below the inclusion threshold and is shown separately as a star marker; myocarditis events occupy the middle portion with substantial age skew toward ≥75.

**Figure 4:**
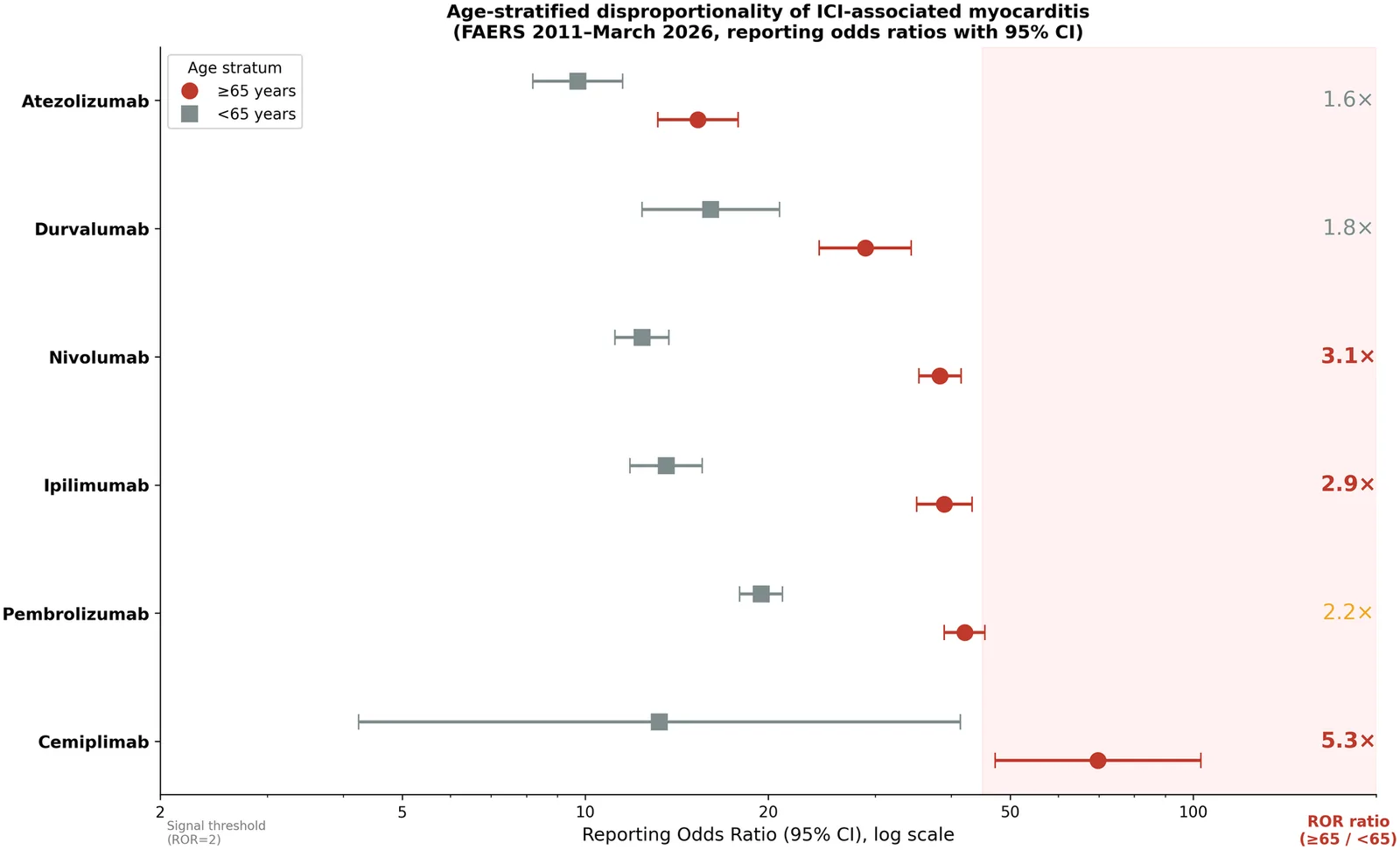
Age-stratified disproportionality of ICI-associated myocarditis (FAERS, 2011–march 2026). Forest plot of reporting odds ratios (ROR) with 95% confidence intervals on a logarithmic scale. Red circles = patients aged ≥65 years; gray squares = patients aged <65 years. Right column shows the fold-amplification ratio (≥65/<65), with values ≥2.5 in bold red. Pink shaded area marks ROR ≥40. Among the five agents with adequate numbers in both strata the amplification ranges from 1.6-fold (atezolizumab) to 3.1-fold (nivolumab). Cemiplimab shows the numerically largest ratio (5.3-fold), but its <65 stratum contains only 3 reports, which is why its confidence interval is the widest in the plot; that ratio is not a stable estimate and should not be read as the upper end of the range. Signal threshold (ROR=2) marked by dotted vertical line.

### 2.2 Statistical analysis

Descriptive analyses summarized total report counts, percentage distributions, and proportions of reports with fatal outcome (hereafter fatality proportion) by drug and reaction category. Patient age at event onset (extracted from patient.patientonsetage; unit and range characterization in Supplementary Table S8) was stratified into three groups (<65, 65–74, ≥75 years) following standard geriatric oncology conventions (26). Reporting odds ratios with 95% confidence intervals were calculated for each ICI agent against the entire FAERS background using the standard 2 × 2 disproportionality table (27, 28) with the formula 95% CI = exp[ln(ROR) ± 1.96 × √(1/a + 1/b + 1/c + 1/d)] where a = drug + myocarditis reports (using the OR-combined unique-case query), b = non-drug + myocarditis reports, c = drug + non-myocarditis reports, and d = non-drug + non-myocarditis reports. Age-stratified RORs were calculated separately for the ≥65 and <65 strata using the corresponding age-restricted backgrounds. A signal was deemed positive when the lower bound of the 95% CI exceeded 1 and the point estimate exceeded 2 (29). All analyses were performed in Python 3.10 (NumPy 1.26, pandas 2.1, SciPy 1.11) and figures generated with Matplotlib 3.8.

To address potential sensitivity of the age-stratification findings to the chosen 65-year cutoff, age-stratified RORs were also calculated using cutoffs of 70 and 75 years (Supplementary Table S3). To examine concomitant medication co-reporting as a potential confounder, the patient.drug field of unique myocarditis reports was parsed for ICI agents and three high-risk co-prescription categories (QT-prolonging agents, beta-blockers, and diuretics) using string matching against established medication lists; up to 1,000 reports per age stratum were retrieved, corresponding to the earliest 1,000 reports in each stratum given that the openFDA API returns records in chronological (oldest-first) order by receive date (Supplementary Table S4). Time from earliest ICI start date to event report receipt (a proxy for time-to-onset, hereafter TTO-proxy) (patient.drug.drugstartdate) to event report date (receivedate) was calculated for reports with both fields populated and a clinically plausible interval (0–730 days), and stratified by patient age (Supplementary Table S5).

### 2.3 Reporting and ethics

The study used aggregate, anonymous, publicly available data from the FDA Adverse Event Reporting System (FAERS); no individual patient data or identifiable information were involved. The Hacettepe University Health Sciences Research Ethics Board reviewed the study protocol and granted a formal waiver of ethics approval (decision no. SBA 26/540, reference E-16969557-050.04-00005080458, dated 17 July 2026), confirming that ethics committee review was not required given the anonymous, publicly available nature of the data. The requirement for informed consent was correspondingly waived. Reporting followed the STROBE checklist for observational studies (30). Processed datasets, analysis code, and query specifications are archived at the Open Science Framework (https://doi.org/10.17605/OSF.IO/HUJYT); the raw FAERS data are publicly available at the URL cited in the Methods.

## 3 Results

### 3.1 Pharmacovigilance reporting profile of ICI-associated cardiotoxicity

Between January 2011 and March 2026, 18,261 ICI-associated cardiac adverse event drug–reaction records were retrieved from FAERS across the six ICI agents and 18 reaction terms (Table 1). For myocarditis specifically, the OR-combined unique-case query identified 4,107 reports across the same period. Pembrolizumab accounted for the largest share of cardiac AE records (*n* = 6,632, 36.3%), followed by nivolumab (*n* = 5,904, 32.3%), ipilimumab (*n* = 2,600, 14.2%), atezolizumab (*n* = 1,995, 10.9%), durvalumab (*n* = 1,017, 5.6%), and cemiplimab (*n* = 113, 0.6%); this distribution mirrors relative market shares since 2018 (31). Reaction category contributions to the overall reporting volume were: myocarditis (records, all variants combined) 36.7%, heart failure/cardiotoxicity 20.3%, arrhythmia 16.5%, myocardial infarction 11.7%, pericardial disease 10.2%, and cardiac arrest 4.7%.

Annual reporting rose substantially over the study period (Figure 1). Total ICI cardiac AE records increased from 85 in 2014 to 2,670 in 2024, a 31-fold rise. The increase was sharper for myocarditis specifically: 3 reports in 2014 grew to 1,253 in 2024 (418-fold). Across all six agents, the interval between first reported case and current peak ranged from 9–12 years, and no agent showed a plateau in annual reporting through 2024.

### 3.2 Disproportionality of ICI-associated myocarditis

Across the full FAERS background of approximately 19.4 million reports during the study period, all six ICI agents showed highly elevated disproportionality signals for myocarditis (Table 3, Figure 4). The pooled ROR for myocarditis across all ICIs combined was 34.21 (95% CI 32.95–35.51), well above the conventional signal threshold and in line with the magnitude reported by Reeves et al. (20) for the 2012–2023 window (ROR 30.1, 95% CI 28.4–32.0). Drug-specific RORs were: pembrolizumab 33.34 (31.74–35.02), ipilimumab 29.36 (27.30–31.58), nivolumab 27.33 (25.90–28.83), durvalumab 26.85 (23.99–30.05), atezolizumab 18.73 (17.05–20.57), and cemiplimab 41.99 (31.76–55.50). The cemiplimab estimate rests on 51 unique myocarditis reports and its confidence interval is correspondingly wide; although this case count is substantially larger than the 7 cases available to Reeves and colleagues, the estimate is reported for completeness rather than as a stable agent-level value, and the ranking of cemiplimab above the other agents should not be read as an established ordering.

Age stratification revealed a feature of the disproportionality signal not previously reported. RORs in the ≥65-year stratum exceeded those in the <65-year stratum for all six ICI agents (Figure 4). Among the five agents with adequate numbers in both strata the amplification ranged from 1.6- to 3.1-fold: nivolumab 3.1-fold (ROR 12.39 in <65 versus 38.32 in ≥65), ipilimumab 2.9-fold (13.58 versus 38.96), pembrolizumab 2.2-fold (19.46 versus 42.07) despite being the most heavily reported agent overall, durvalumab 1.8-fold (16.08 versus 28.87), and atezolizumab 1.6-fold (9.72 versus 15.32); atezolizumab also yielded the lowest absolute ROR in the ≥65 stratum (15.32, 95% CI 13.16–17.85). Cemiplimab returned the numerically largest ratio, 5.3-fold (13.24, 95% CI 4.24–41.38, versus 69.71, 47.20–102.96), but only 3 cemiplimab reports fell in the <65 stratum. A ratio computed against a three-report denominator is not a stable estimate of amplification, as the width of that confidence interval shows, and it is therefore excluded from the range quoted above and reported separately throughout.

The age concentration of myocarditis reports does not simply reflect the fact that older patients contribute a larger share of ICI-related reports overall: their reporting odds for myocarditis within age-stratified FAERS reporting backgrounds are also disproportionately higher than those of younger patients. The pattern is most pronounced for the agents with the highest myocarditis fatality proportions.

### 3.3 ICI-associated myocarditis is a geriatric problem: age structure of affected patients

Among 3,996 unique myocarditis reports with recorded patient age (Table 2 and Figure 2), 36.6% occurred in patients aged <65 years, 35.2% in those aged 65–74, and 28.3% in those aged ≥75. Two-thirds (63.4%) of all ICI myocarditis reports therefore occurred in patients aged 65 or older, and over a quarter occurred in patients aged 75 or older. These proportions exceed the participation rates of these age groups in the pivotal trials of each agent by 10.4–25.7 percentage points (Supplementary Table S6) (6). The age distribution differed substantially across agents. Pembrolizumab and atezolizumab showed the most balanced distributions (≥65: 59.0% and 56.4% respectively); nivolumab (68.0%), ipilimumab (66.0%), and durvalumab (69.9%) showed greater concentration in older patients. Cemiplimab, on 30 age-recorded reports and therefore on the least stable denominator of the six agents, showed the most pronounced age skew in the reporting pattern (90.0% of myocarditis reports in patients aged ≥65 and 53.3% in those aged ≥75) — a pattern consistent with its primary indication, cutaneous squamous cell carcinoma, which is a disease of advanced age (32).

The age skew was not confined to myocarditis. Across non-myocarditis cardiac event categories, the proportion of records in patients aged ≥75 ranged from 10% (pericarditis with ipilimumab) to 39% (atrial fibrillation with atezolizumab). Atrioventricular block complete records were particularly concentrated in older adults, with 35%–39% in the ≥75 stratum across pembrolizumab, nivolumab, and ipilimumab, exceeding the proportion observed for myocarditis with the same agents and consistent with the high prevalence of underlying conduction system disease and infiltrative fibrosis of the conduction tissue described in cardiac aging (15).

### 3.4 Mortality and the convergence of age and lethality

Across all 4,107 unique ICI myocarditis reports, 1,343 (32.7%) had an outcome of death. Proportions of reports with fatal outcome, by agent, were: pembrolizumab 31.4%, nivolumab 35.5%, ipilimumab 33.4%, atezolizumab 26.9%, durvalumab 33.9%, and cemiplimab 49.0%. The pooled estimate is in line with the 37% rate reported by Reeves et al. (20) and reflects the gradual improvement in outcomes over the past decade as recognition and treatment have advanced. Beyond the myocarditis unique-case analyses above, the drug-reaction record analyses (Table 1) showed that cardiac arrest events demonstrated the highest case-fatality rates of any reaction category, ranging from 76.3% (nivolumab) to 81.6% (atezolizumab) across the five agents with adequate sample sizes (*n* = 857 records across five agents; pooled fatality 78.1%); cemiplimab reported 7 of 7 cardiac arrest events as fatal (100%), a rate that cannot be reliably interpreted given the very small denominator. Ventricular fibrillation reports also showed high mortality (50.6% pooled across agents). When mortality and age skew were examined jointly (Figure 3 and Supplementary Table S2 and Supplementary Figure S1), the reaction categories with the highest fatality rates also showed higher proportions of records in patients aged ≥75. A similar convergence is apparent for cemiplimab, whose 30 age-recorded reports place 90.0% of cases in patients aged ≥65 alongside the highest fatal-outcome proportion of any agent (49.0%); with denominators of this size neither figure is a stable estimate, and the co-occurrence is noted rather than quantified.

## 4 Discussion

It is important to emphasize that ROR values derived from FAERS reflect reporting disproportionality within a spontaneous adverse event database and do not represent true clinical incidence or direct relative risk in the general population. Because FAERS does not provide true exposure denominators by age, treatment duration, indication, tumor type, ICI combination regimen, or comorbidity status, the age-stratified reporting odds ratios reported here should be interpreted as age-stratified reporting signals rather than as direct evidence that older age independently increases the true clinical risk of ICI myocarditis. The estimates below are hypothesis-generating signals requiring confirmation in exposure-controlled cohort studies.

With this caveat in mind, and to keep demonstrated findings clearly separated from biological hypotheses and clinical implications, the remainder of this discussion is organized in three layers: (i) findings directly demonstrated by our FAERS data (age concentration of myocarditis reports, age-stratified reporting odds ratios, fatality proportions, and temporal reporting patterns); (ii) biological and structural mechanisms that plausibly explain these reporting patterns but are not themselves demonstrated by our data (immunosenescence, inflammaging, cardiac reserve, atypical clinical presentation, polypharmacy); and (iii) clinical implications that follow if (i) and (ii) hold (pre-ICI geriatric-cardiac evaluation, structured surveillance, patient and caregiver communication).

This analysis shows that ICI-associated cardiotoxicity, as captured in spontaneous reporting, is concentrated in older adults: two-thirds of myocarditis reports with known age occurred in patients aged ≥65, and over a quarter in patients aged ≥75. These proportions exceed the participation rates of these age groups in the pivotal trials that established ICI safety profiles by 10.4–25.7 percentage points (6). The most extreme age skew, for cemiplimab (90.0% in ≥65 and 53.3% in ≥75, across 30 age-recorded reports), is close to what its indication alone would predict: cutaneous squamous cell carcinoma is a disease of advanced age and the population enrolled in the cemiplimab registration programme was correspondingly old (32), so a reporting population drawn almost entirely from older patients follows from who receives the drug rather than from any agent-specific age effect.

A more novel observation is that the pharmacovigilance disproportionality signal itself varies with age. The higher reporting odds ratios in older patients do not merely reflect a larger share of ICI-related reports overall in this group; their reporting odds for myocarditis within age-stratified FAERS reporting backgrounds are higher than those of younger patients for every agent examined, by 1.6- to 3.1-fold among the five agents with adequate numbers in both strata. The numerically largest ratio, cemiplimab's 5.3-fold, is computed against three younger reports and cannot be read as the upper end of that range. Nivolumab and ipilimumab, the agents most often used in combination, where the myocarditis reporting signal is highest (33, 34), both show close to threefold age amplification on substantially larger denominators. To our knowledge, no previous FAERS analysis has demonstrated that the disproportionality signal for ICI myocarditis varies meaningfully by patient age, and the pattern complements rather than duplicates earlier overall analyses, which did not stratify by age.

Cemiplimab requires separate comment, and a cautious one. Its 51 myocarditis reports, of which 30 carry a recorded age and only 3 fall in the <65 stratum, are the smallest evidence base of any agent in this analysis; the 95% CI for its <65 ROR spans an order of magnitude (4.24–41.38), and no estimate built on that denominator should be treated as stable. With that stated first, three features coincide for this agent: the highest proportion of reports with fatal outcome (49.0%), the most age-concentrated reporting pattern (90.0% aged ≥65), and the highest absolute reporting odds ratio in the ≥65 stratum. Two explanations must be distinguished before any of this is read as an agent-specific geriatric hazard. The first is indication: cutaneous squamous cell carcinoma, cemiplimab's principal approved indication, occurs overwhelmingly in older adults, so a reporting pattern concentrated in that group is what the treated population alone would produce. The second would be a genuine amplification of cardiotoxic risk in older patients over and above what the treated population predicts. Our data cannot separate these, because FAERS provides no exposure denominators by indication or age; and the indication explanation is sufficient to account for the observed age skew without invoking the second. Cemiplimab therefore warrants attention as a hypothesis for larger, exposure-controlled datasets rather than as an agent for which a distinct geriatric warning is presently supported.

These findings have direct relevance for cancer care in countries with rapidly aging populations. Türkiye illustrates this concern. In 2020, 233,834 new cancer cases were diagnosed (35), and the population aged ≥65 was projected to exceed 12% by 2025 and to approximately double by 2050. ICI access has also expanded under reimbursement by the SGK (Sosyal Güvenlik Kurumu; Social Security Institution of Türkiye) since the late 2010s. The geriatric concentration we describe in FAERS is therefore likely to be reflected in domestic real-world practice. In many middle-income settings, ICI use has expanded faster than the geriatric and cardio-oncology infrastructure that should support it. Türkiye exemplifies this pattern: geriatric oncology consultation is concentrated in academic medical centers while ICI prescribing has diffused into community oncology practice, so structured pre-treatment geriatric–cardiac evaluation faces real workflow constraints. Coordinated pathways between geriatrics, cardiology, and medical oncology services are likely to be needed before this framework can be integrated into routine care in similar settings. FAERS receives a substantial proportion of its reports from outside the United States, so the geriatric concentration we describe is unlikely to be a purely US phenomenon and is likely to be relevant wherever ICI use is expanding into an aging population.

### 4.1 Why is ICI cardiotoxicity concentrated in older adults? a geriatric framework

The most consequential observation in this analysis is not that older adults account for most ICI myocarditis reports; that follows in part from who receives these drugs. It is that their reporting odds for myocarditis are elevated relative to their own age-matched reporting background. Because each stratum is compared with the FAERS background for the same stratum, the amplification is not generated by the larger overall report volume that older patients produce, nor by their greater exposure to medication and to reporting in general. What the 1.6- to 3.1-fold amplification describes is a change in the composition of what is reported for older patients: within everything reported about this age group, myocarditis occupies a larger share than it does among younger patients. The direction is consistent across every agent examined and survives moving the age cutoff to 70 and to 75 years (Supplementary Table S3).

Two explanations are compatible with this pattern, and the present data cannot separate them. The first is that the true incidence of ICI myocarditis rises with age, which would be consistent with the immunological and structural changes set out below. The second is that events of comparable true incidence are more often recognised, judged serious, and reported in older patients, because the same degree of immune-mediated myocardial injury is less well tolerated against reduced cardiac reserve and therefore declares itself more severely. These readings differ in what they imply about aetiology but converge clinically: on either account, an older patient who develops ICI myocarditis is more likely to come to attention as a serious event. The practical corollary is that agent-level safety information derived from unstratified analyses is a poor guide to what to expect in an older patient. For five of the six agents the ≥65 estimate exceeds that agent's own unstratified ROR (atezolizumab is the exception), and for all six the <65 estimate falls below it; the pooled figure that reaches the clinician therefore sits above the reporting profile of the younger patient and below that of the older one.

Our pharmacovigilance findings are consistent with the only dedicated real-world geriatric ICI cardiotoxicity dataset published to date. Toribio-García and colleagues (24) followed 195 patients aged ≥70 (mean age 75; 76.9% male) treated with ICIs at the University Hospital of Salamanca and identified three immune-related cardiac events (1.54% incidence), of which two-thirds were myocarditis and one-third arrhythmias, with median time-to-event of 45 days from ICI initiation. The myocarditis-predominant pattern they observed mirrors the FAERS reaction-category distribution we report, and their median time-to-event aligns with the three-month high-risk window after ICI initiation noted in our discussion of clinical surveillance. Their cohort was too small for stratified disproportionality analysis, but the demographic concentration of cardiac events they observed in patients with mean age 75 is congruent with our age-amplified ROR signal. Taken together, the pharmacovigilance signal and the available real-world evidence point to the same conclusion: ICI cardiotoxicity in older adults is neither rare nor evenly distributed across age, and current surveillance approaches have not been adapted to this distribution.

Several mechanisms plausibly contribute to the geriatric concentration of ICI myocarditis we observed. An immunological contribution arises from age-related changes in T-cell biology. Aging is accompanied by reduced thymic output, contraction of the naïve T-cell repertoire, expansion of clonal CD8 + effector populations, and a chronic low-grade inflammatory state often described as inflammaging (16, 17). These changes shift peripheral immune tolerance and may render the aged myocardium more vulnerable to T-cell-mediated injury once checkpoint blockade releases its molecular brakes. This mechanism is not demonstrated by our data; it is offered as one plausible account of a reporting pattern that trial-derived safety profiles, drawn from younger cohorts, would not have surfaced.

A second, structural contribution arises from progressive changes in the aging heart itself. The aging cardiovascular system shows collagen deposition, myocardial fibrosis, sinoatrial pacemaker cell loss, and conduction system stiffening (15). These changes lower the threshold at which any acute myocardial insult — autoimmune or otherwise — translates into clinically detectable injury, arrhythmia, or hemodynamic compromise. The high prevalence of complete atrioventricular block records in the ≥75 stratum (35%–39% across the major agents) is consistent with this background substrate: an immune-mediated insult superimposed on already vulnerable conduction tissue.

A third contribution lies in the difficulty of clinical recognition. Older adults frequently present with atypical or attenuated symptoms such as non-specific fatigue, exertional intolerance, vague chest discomfort, or mild dyspnea, which overlap with chronic comorbidity. Concurrent immune-mediated myositis or myasthenic syndromes further complicate recognition in approximately 25% of ICI myocarditis cases (18, 36). Polypharmacy may further obscure the picture: QT-prolonging psychotropics, beta-blockers attenuating tachycardia, and diuretics affecting volume status could hypothetically mask early signs of myocarditis. Frailty itself reduces the patient's capacity to articulate functional decline as a discrete change. The result is that the threshold for considering ICI myocarditis is too often raised by precisely the comorbidity burden that should lower it.

Two further analyses support this geriatric framework. The concomitant medication co-reporting in unique ICI myocarditis reports increased monotonically with age: among patients aged ≥75, 17.0% had a co-prescribed QT-prolonging agent, beta-blocker, or diuretic, compared with 9.7% in patients aged <65 (Supplementary Table S4). Beta-blocker co-prescription was 2.5-fold higher (10.4% vs. 4.2%) and diuretic co-prescription 3.2-fold higher (9.0% vs. 2.8%) in the ≥75 stratum. These agents may attenuate compensatory tachycardia and alter volume status during early myocarditis, plausibly delaying clinical recognition. In parallel, the interval from earliest recorded ICI start date to FAERS report receipt (a proxy for time-to-onset, since the true clinical event date is not captured in FAERS) was substantially shorter in older patients: median 97 days (Q1–Q3: 53–189) in those aged ≥75 versus 148 days (62–259) in those aged <65. Nearly half of older patients (47.2% of ≥75) had their myocarditis reports received within 90 days of recorded ICI start, compared with 37.8% of younger patients (this metric reflects reporting latency plus true event onset and should be interpreted as a temporal reporting pattern rather than as true clinical onset) (Supplementary Table S5). The earlier report receipt in older adults is biologically consistent with the structural and immunological mechanisms outlined above and reinforces the clinical importance of intensified surveillance during the first three months of ICI therapy in geriatric oncology practice.

Read alongside the shorter interval to report receipt and the higher burden of cardioactive co-medication in the oldest stratum, the age-stratified signal argues for surveillance that is front-loaded rather than uniform across the treatment course. The window in which nearly half of the reports concerning patients aged ≥75 arrive is the first three months, and it is the same window in which atypical presentation, blunted compensatory tachycardia under beta-blockade, and competing explanations for fatigue and dyspnoea are most likely to divert attention from the diagnosis. Chronological age is a crude trigger for intensified monitoring, and a better one would incorporate cardiac reserve and geriatric domain variables that FAERS does not capture. On present evidence, however, it is the trigger these data support: it identifies the group in which the myocarditis share of reported events is largest, the interval to report receipt shortest, and the burden of cardioactive co-medication highest.

### 4.2 A conceptual pre-ICI geriatric-cardiac evaluation framework for older adults

Our findings strengthen the case for incorporating cardiac evaluation into the pre-ICI workup for older patients receiving any agent of this class. The age amplification we observed is present for every agent examined, so the case for pre-treatment assessment rests on the patient's age rather than on the choice of agent; the between-agent differences in our data are too exposed to differences in indication, combination use and report volume to justify an agent-specific pathway. The 2022 ESC cardio-oncology guideline (14) already recommends baseline electrocardiography and high-sensitivity troponin assessment before ICI initiation. We propose extending this for adults aged ≥65, and especially ≥75, by anchoring it within the Comprehensive Geriatric Assessment (CGA) framework already established for chemotherapy tolerance (26, 37).

A conceptual pre-ICI geriatric–cardiac framework (Figure 5) would include four domains: (i) a brief geriatric screen using a validated tool such as the G8 instrument (sensitivity 85%, specificity 65% at the cut-off of 14 for triggering full CGA in cancer patients aged ≥70) (38), to identify patients who warrant closer surveillance during the high-risk first three months. A G8 score ≤14 is used in existing guidance to identify patients for full CGA, ideally including basic and instrumental activities of daily living (Katz ADL, Lawton IADL), cognitive screening [Mini-Mental State Examination [MMSE] or Montreal Cognitive Assessment [MoCA]], mobility and falls risk (Tinetti or Timed Up and Go), mood (GDS-15), and nutritional status (MNA-SF), in line with International Society of Geriatric Oncology (SIOG) recommendations for chemotherapy tolerance assessment (37). (ii) An objective cardiovascular reserve assessment combining baseline ECG [with attention to QRS duration, which is prognostic in ICI myocarditis (39)], high-sensitivity troponin, and brief physical performance measures (gait speed, grip strength) that correlate with cardiovascular reserve and frailty. (iii) A structured polypharmacy review focused on QT-prolonging agents, rate-modifying drugs that may mask myocarditis-related tachycardia, and medications interacting with cardiac autonomic tone. (iv) Explicit pre-treatment counseling delivered to the patient and an identified caregiver, framing the warning symptoms in plain language and providing a low-threshold contact route. Dermatologic-oncology clinics, where patients treated for cutaneous squamous cell carcinoma are typically old and pre-ICI cardiac assessment is currently inconsistent, are one setting in which such a pathway could be implemented; this reflects the age of the population treated in that setting rather than any agent-specific risk established by our data.

**Figure 5:**
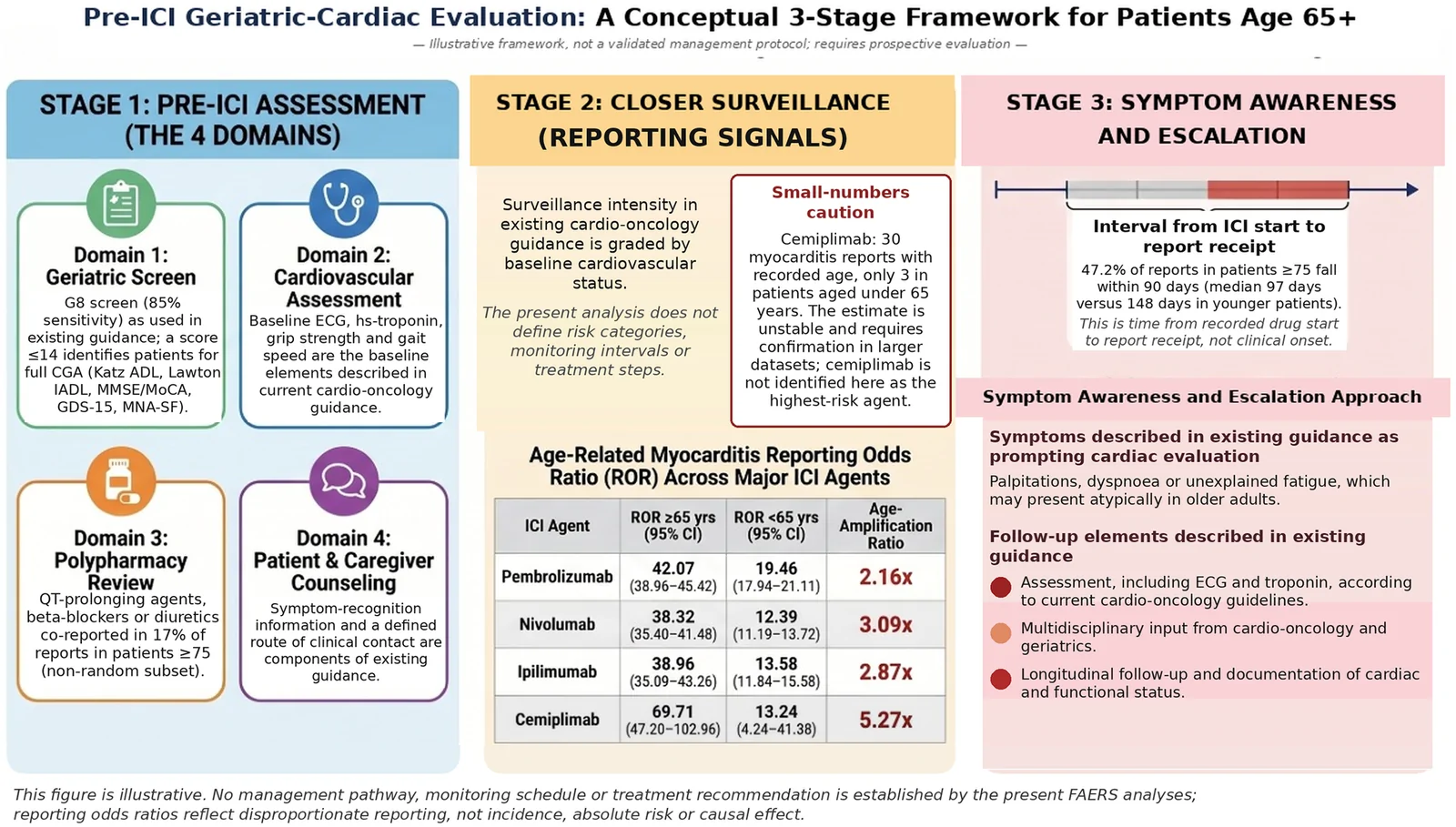
Conceptual three-stage pre-ICI geriatric–cardiac evaluation framework (illustrative, not prescriptive) for patients aged ≥65 receiving immune checkpoint inhibitors. Stage 1 sets out four parallel pre-ICI assessment domains drawn from existing guidance: geriatric screening with the G8 instrument (85% sensitivity), a score ≤14 identifying patients for full comprehensive geriatric assessment; baseline cardiovascular assessment (12-lead ECG, high-sensitivity troponin, grip strength and gait speed); structured polypharmacy review (QT-prolonging agents, beta-blockers or diuretics were co-reported in 17% of reports in patients aged ≥75, a non-random subset); and patient and caregiver counselling on symptom recognition and route of contact. Stage 2 displays the age-stratified reporting odds ratios on which the framework draws. Surveillance intensity in existing cardio-oncology guidance is graded by baseline cardiovascular status, and the present analysis does not define risk categories, monitoring intervals or treatment steps; no agent-specific tier adjustment is proposed. The small-numbers caution box records that the cemiplimab estimate rests on 30 age-recorded reports with only 3 in the <65 stratum, is unstable, requires confirmation in larger datasets, and does not identify cemiplimab as the highest-risk agent. Stage 3 shows the interval from recorded ICI start to report receipt — 47.2% of reports in patients aged ≥75 fall within 90 days, median 97 days versus 148 days in younger patients, which is a reporting interval and not a clinical onset — alongside the symptoms and follow-up elements described in existing guidance (14). Reporting odds ratios reflect disproportionate reporting, not incidence, absolute risk or causal effect. The framework is adapted from ESC 2022 cardio-oncology recommendations and SIOG geriatric-oncology principles; it is not a validated management protocol, and the specific age cutoffs, screening intervals and thresholds are illustrative and require prospective evaluation.

This approach would not replace the ESC framework but would focus its delivery on the patient group in whom the signal is most concentrated. Given that the ESC guidelines already recommend baseline cardiac evaluation for all patients receiving ICIs, the practical priority is how best to deliver closer attention to older adults within the workflow constraints of geriatric oncology practice.

A supplementary analysis of ICI–ICI and chemotherapy co-reporting within the unique myocarditis reports (Supplementary Table S7; these analyses included reports carrying any of the three myocarditis Preferred Terms, so per-agent denominators exceed those in Table 1) showed that 94.1% of ipilimumab reports (769 of 817) and 49.5% of nivolumab reports (791 of 1,598) also named a second ICI agent; in both cases the partner was almost always the other agent of the pair (759 reports naming both nivolumab and ipilimumab). Drug-specific reporting odds ratios for these two agents therefore represent combined signals rather than pure monotherapy signals. Co-listing of a second ICI was uncommon for pembrolizumab (5.2%) and cemiplimab (1.8%), but was not negligible for atezolizumab (17.1%) or durvalumab (17.3%). Restricting to reports without a co-listed second ICI would leave 807 nivolumab and only 48 ipilimumab myocarditis reports, so a monotherapy-restricted ipilimumab estimate would be too imprecise to interpret. Because dual-agent ICI therapy is itself a well-recognized risk factor for myocarditis (34), a monotherapy-restricted analysis would be expected to yield lower drug-specific reporting odds ratios for both agents but would not eliminate the class-wide myocarditis signal or the age-related concentration of that signal, both of which rest most robustly on pembrolizumab (1,931 reports without a co-listed ICI) and cemiplimab (56 reports; effectively monotherapy). Concomitant cardiotoxic chemotherapy (anthracyclines, fluoropyrimidines, or platinum compounds) was co-reported in a minority of myocarditis reports for every agent (5.6% to 28.0%), and this co-reporting was overwhelmingly platinum-based (84% to 100% of the co-reporting for each agent), consistent with the routine use of platinum-containing chemo-immunotherapy regimens rather than with exposure to the chemotherapy classes most strongly linked to myocarditis; anthracycline co-reporting was uncommon throughout (0.0% to 6.6%). Only 8.8% of cemiplimab myocarditis reports named any cardiotoxic chemotherapy and none named an anthracycline, so the cemiplimab age skew is not attributable to chemotherapy co-exposure. Co-listing indicates that two agents appear in the same case record, not that they were administered concurrently; reports naming two PD-1/PD-L1 agents (for example pembrolizumab and atezolizumab, 72 reports) most plausibly reflect sequential lines of therapy. These combination-therapy patterns do not overturn the primary findings but do warrant caution in interpreting drug-specific reporting odds ratios for nivolumab and ipilimumab in isolation. Furthermore, chemotherapy use in the ICI population is well known to decrease with age [fitter, younger patients receive more intensive chemo-immunotherapy regimens, whereas older patients more often receive single-agent PD-(L)1 blockade], a pattern that would attenuate rather than amplify any chemotherapy-driven contribution to the geriatric myocarditis signal we describe.

Combination regimens pairing an ICI with a tyrosine kinase inhibitor are now standard of care in several of the indications covered by these agents, including pembrolizumab with lenvatinib or axitinib and nivolumab with cabozantinib in renal cell and endometrial carcinoma. Tyrosine kinase inhibitors carry a recognised cardiotoxicity profile of their own — hypertension, QT prolongation and cardiomyopathy — so a myocarditis or heart-failure report naming an ICI may reflect TKI-related cardiac injury attributed to the immunotherapy, or an interaction between the two drug classes. Applying the string-matching approach used above for chemotherapy to a defined list of TKIs used in approved ICI combinations (lenvatinib, cabozantinib, axitinib), co-reporting within unique myocarditis reports ranged from 0% (cemiplimab, 0 of 57) to 13.8% (pembrolizumab, 280 of 2,036), with nivolumab at 3.4% (54 of 1,598), ipilimumab 3.3% (27 of 817), atezolizumab 1.7% (8 of 480) and durvalumab 1.2% (4 of 347) (Supplementary Table S7c). The partner agent tracked the approved combinations: pembrolizumab co-reporting was driven by lenvatinib, named in 204 reports, and axitinib, named in 78; nivolumab and ipilimumab co-reporting was almost entirely cabozantinib, named in 44 and 23 reports respectively. Broadening the list to any VEGFR-directed or multikinase TKI barely changed these proportions (13.8%, 3.6%, 3.4%, 2.7%, 1.4% and 0% respectively), indicating that TKI co-exposure in these reports is concentrated in the approved combination regimens rather than distributed across kinase inhibitor use generally. TKI co-reporting does not track the age gradient across agents: pembrolizumab, with by far the highest proportion, shows one of the smaller age amplifications (2.2-fold), while cemiplimab, with no TKI co-reporting at all, shows the largest; with six agents this is an observation rather than a formal test, but it does not support TKI co-exposure as an explanation for the age-stratified pattern. For pembrolizumab, however, TKI co-listing (13.8%) is more common than co-listing of a second ICI (5.2%), so it is a material component of the treatment context behind that agent's drug-specific estimate. This co-exposure is distinct from, and additional to, the ICI–ICI and ICI–chemotherapy co-reporting described above, and applies to a partly different set of agents and indications. As with the other co-reporting layers, co-listing establishes only that two drugs appear in the same case record; concurrent administration cannot be verified in FAERS, so these proportions bound the possible contribution of TKI co-exposure rather than measure it.

The counseling domain of the proposed framework does not rest on our reporting data but on the recognition problem described in Section 4.1: when ICI myocarditis presents in an older adult as fatigue, reduced exercise tolerance, or vague chest discomfort rather than as classical chest pain, the patient's own interpretation of those symptoms becomes part of the diagnostic pathway. Pre-treatment counseling that names these symptoms explicitly, is calibrated to an appropriate reading level (40), and is directed to an identified family caregiver as well as to the patient is therefore a component of the proposed workflow rather than an optional addition. Whether such counseling shortens time to presentation in older patients has not been tested and requires prospective evaluation.

### 4.3 Limitations

FAERS is a spontaneous reporting system: case ascertainment is incomplete, denominators are unknown, and reporting rates vary with drug familiarity, indication expansion, regulatory attention, and clinician awareness (41). Apparent rises in reporting cannot be cleanly attributed to rising true incidence versus rising recognition or reporting fidelity. Our age-stratified RORs are protected against differential exposure between age groups by design, because they compare reporting odds within each stratum to the same-stratum background. Residual confounding from age-related differences in indication mix, comorbidity, and reporting behavior nevertheless remains possible. Proportions of reports with fatal outcome derived from FAERS are subject to over-representation of severe outcomes because mild events are less often reported.

Per-agent age completeness of the unique myocarditis reports ranged from 59% (cemiplimab) to 84% (pembrolizumab); overall age availability across the 18,261 ICI cardiac adverse event records was 79.5% (14,516 records with a recorded age value; Supplementary Table S8). Reports naming two ICI agents are attributed to both, so the pooled age figures are computed over the sum of per-agent counts rather than over the de-duplicated pooled total. Sensitivity analysis across the full range of de-duplication assumptions yields ≥65 proportions between 52.1% and 83.1%, with a plausible range of 62.6–63.4% (Supplementary Table S1); the qualitative conclusion that ICI myocarditis reporting is concentrated in older adults holds throughout. Cross-category comparisons in Table 1 use drug–reaction record counts; reports containing more than one Preferred Term within the same category are counted once per term, so absolute totals across categories should be interpreted as records, not unique cases. Myocarditis-specific results (Sections 3.2–3.4, Tables 2, 3, Figures 2, 3, 4) use unique-report counts: the pooled total via an OR-combined query across the three myocarditis Preferred Terms, and the per-agent counts via the primary “myocarditis” Preferred Term.

Three distinct co-exposure confounders therefore bear on the drug-specific reporting odds ratios in this analysis: co-listing of a second ICI, co-listing of cytotoxic chemotherapy, and co-listing of a tyrosine kinase inhibitor (Supplementary Tables S7a–S7c). Each is documented in Section 4.2 and none can be resolved within FAERS, which records the drugs named in a case but neither their timing nor their causal attribution. The drug-specific estimates should therefore be read as signals attached to a treatment context rather than to a single molecule; the age-stratified comparison, which holds the treatment context approximately constant within each stratum, is less exposed to this limitation than the between-agent comparison.

The proposed pre-ICI geriatric–cardiac framework described in Section 4.2 is conceptual, derived from our pharmacovigilance findings combined with existing CGA literature; it has not been prospectively evaluated and requires implementation studies before adoption into routine practice. FAERS does not capture geriatric domain variables that are likely to modify ICI cardiotoxicity outcomes, such as frailty status, sarcopenia, baseline cognitive function, falls history, or geriatric syndromes such as delirium, which may serve as the first manifestation of myocarditis in older adults with attenuated chest symptoms. Prospective cohorts integrating CGA components with cardiac biomarkers are needed to refine pre-treatment risk stratification beyond chronological age.

Sensitivity analyses with age cutoffs of 70 and 75 (Supplementary Table S3) and with the analytic cohort restricted to age 18-110 years recorded in year units (Supplementary Table S8) confirm that the age-amplified ROR pattern is robust to cutoff and to age-quality restrictions for five of six ICI agents. The magnitude of cemiplimab's amplification is sensitive to the chosen cutoff because of the small number of younger comparators (only 8 cemiplimab myocarditis cases were aged <70). This limitation does not negate the high absolute ROR observed for cemiplimab in older adults, which remains the highest of any agent at every cutoff.

## 5 Conclusion

Across 4,107 unique ICI-associated myocarditis reports filed to FAERS over 15 years, ICI myocarditis is disproportionately concentrated in older adults. Two-thirds of cases occur in patients aged ≥65, and the disproportionality signal itself is amplified in this age group for every agent examined — 1.6- to 3.1-fold among the five agents with adequate numbers in both strata, with a larger but unstable ratio for cemiplimab, whose comparison stratum contains three reports. Older adults receiving immunotherapy therefore appear disadvantaged on two fronts of the ICI evidence landscape: under-representation in the trials that established the safety profile of this class, and an age-amplified pharmacovigilance signal concentrated in precisely the group that those trials studied least. Integrating cardiac evaluation into geriatric oncology practice is a practical next step for cardio-oncology and geriatric oncology as ICI use expands further into older populations. Such integration should use CGA principles, focus on the age strata at highest risk, and include caregiver-directed communication.

## Data Availability

Publicly available datasets were analyzed in this study. FAERS data are publicly available through the openFDA API (https://api.fda.gov/drug/event.json) and the FDA FAERS public dashboard (https://fis.fda.gov/sense/app/95239e26-e0be-42d9-a960-9a5f7f1c25ee/overview). Processed datasets, analysis code and query specifications are archived at the Open Science Framework (https://doi.org/10.17605/OSF.IO/HUJYT).
