# Supplementary material for: Age-stratified reporting of immune checkpoint inhibitor-associated myocarditis: disproportionality is amplified in older adults, with implications for geriatric oncology
Source: Front Cardiovasc Med. 2026 Oct 13;13:1948161. doi: 10.3389/fcvm.2026.1948161 (PMC13709986; doi:10.3389/fcvm.2026.1948161)
Supplement: Supplementary file 1 [file Datasheet1.docx]

**Supplementary Material**

*Tables S1–S8, Figure S1 and Supplementary Methods S1.*

**Table S1. Sensitivity analysis for de-duplication of combination-therapy reports in unique ICI myocarditis reports.**

| **Scenario** | **≥65 years proportion (%)** |
| --- | --- |
| **Lower bound: all dual-listed reports had age recorded and were aged ≥65** | 52.1% |
| **Plausible range: dual-listed reports aged as their component agents** | 62.6% – 62.7% |
| **As reported: per-agent sum without de-duplication** | 63.4% |
| **Upper bound: all dual-listed reports had age recorded and were aged <65** | 83.1% |

*Per-agent age completeness of the unique myocarditis reports ranged from 59% (cemiplimab) to 84% (pembrolizumab); overall age availability across the 18,261 cardiac adverse event records was 79.5% (Supplementary Table S8). Sensitivity of the pooled ≥65 proportion to de-duplication of combination-therapy reports. The six per-agent unique myocarditis counts sum to 5,053 against 4,107 de-duplicated pooled reports, so 946 reports name two ICI agents and are counted twice in the per-agent age sums. The extreme bounds assume all such reports fall entirely into one age stratum. Because these reports are predominantly nivolumab plus ipilimumab, and 66–68% of reports for both agents are in patients aged ≥65, the plausible range is narrow and close to the reported estimate. The qualitative conclusion, that ICI myocarditis reporting is concentrated in older adults, holds across the full bracket.*

**Table S2. ICI cardiac adverse events by reaction category, with proportion of reports with fatal outcome (FAERS, 2011–March 2026).**

| **Reaction category** | **Total reports (n)** | **Fatal reports (n)** | **Fatal reports (%)** |
| --- | --- | --- | --- |
| **Myocarditis (unique cases)** | 4,107 | 1,343 | 32.7% |
| **Heart failure / cardiotoxicity (records)** | 3,700 | 1,106 | 29.9% |
| **Arrhythmia (records)** | 3,010 | 772 | 25.7% |
| **Myocardial infarction (records)** | 2,128 | 856 | 40.2% |
| **Pericardial (records)** | 1,865 | 327 | 17.5% |
| **Cardiac arrest** | 864 | 675 | 78.1% |

*Fatality proportion (reports coded as fatal / total reports) calculated from reports with known outcome status; this is a reporting-based fatal-outcome proportion, not a clinical case-fatality rate. FAERS death outcomes do not establish that myocarditis or the cardiac AE caused death, and severe cases are more likely to be reported than mild cases. Cardiac arrest events show the highest proportion of reports with fatal outcome. Myocarditis counts are unique cases (OR-combined query); other categories are drug–reaction records.*

**Table S3. Sensitivity analysis: age-stratified disproportionality of ICI-associated myocarditis using alternative age cutoffs (≥70 and ≥75 years).**

| **ICI agent** | **Cutoff** | **ROR ≥cutoff (95% CI)** | **ROR <cutoff (95% CI)** | **Ratio** |
| --- | --- | --- | --- | --- |
| **Pembrolizumab** | ≥70 | 47.73 (43.65–52.18) | 20.75 (19.33–22.28) | 2.30 |
| **Pembrolizumab** | ≥75 | 60.77 (54.13–68.23) | 22.49 (21.12–23.94) | 2.70 |
| **Nivolumab** | ≥70 | 41.67 (37.96–45.74) | 15.27 (14.07–16.58) | 2.73 |
| **Nivolumab** | ≥75 | 54.94 (48.74–61.93) | 17.14 (15.97–18.40) | 3.21 |
| **Ipilimumab** | ≥70 | 43.39 (38.38–49.05) | 16.36 (14.63–18.30) | 2.65 |
| **Ipilimumab** | ≥75 | 50.12 (42.48–59.14) | 19.33 (17.58–21.25) | 2.59 |
| **Atezolizumab** | ≥70 | 16.32 (13.62–19.56) | 10.25 (8.86–11.85) | 1.59 |
| **Atezolizumab** | ≥75 | 17.24 (13.39–22.20) | 11.04 (9.73–12.53) | 1.56 |
| **Durvalumab** | ≥70 | 33.10 (27.14–40.36) | 16.55 (13.39–20.45) | 2.00 |
| **Durvalumab** | ≥75 | 43.43 (33.65–56.04) | 18.01 (15.11–21.46) | 2.41 |
| **Cemiplimab** | ≥70 | 67.75 (43.99–104.35) | 27.12 (13.43–54.78) | 2.50 |
| **Cemiplimab** | ≥75 | 71.21 (42.91–118.16) | 36.31 (21.29–61.92) | 1.96 |

*Reporting odds ratios calculated using the standard 2×2 disproportionality table against age-restricted FAERS backgrounds. Comparison with the primary 65-year cutoff (Table 3): for five of six agents (pembrolizumab, nivolumab, durvalumab, atezolizumab, ipilimumab) the amplification pattern is preserved or strengthened with higher cutoffs. For cemiplimab, the amplification ratio decreases with higher cutoff because of the small number of cases in younger strata (n=8 at <70; n=14 at <75); the absolute ROR in older adults remains the highest of any agent at every cutoff.*

**Table S4. Concomitant medication co-reporting in unique ICI-associated myocarditis reports, stratified by patient age.**

| **Age stratum** | **Sample size** | **Mean conc. drugs** | **Median** | **QT-prolong. (%)** | **β-blocker (%)** | **Diuretic (%)** | **Any high-risk (%)** |
| --- | --- | --- | --- | --- | --- | --- | --- |
| **<65 yrs** | 1,000 | 4.5 | 3 | 4.3% | 4.2% | 2.8% | 9.7% |
| **65–74 yrs** | 1,000 | 5.2 | 3 | 3.9% | 8.0% | 7.4% | 14.2% |
| **≥75 yrs** | 969 | 4.6 | 2 | 3.3% | 10.4% | 9.0% | 17.0% |

*Sample size reflects the number of unique myocarditis reports parsed for concomitant drugs, capped at 1,000 per age stratum by the openFDA pagination limit. Records were returned in chronological (oldest-first) order by receive date and were therefore not randomly sampled; the proportions below should be read as descriptive and hypothesis-generating. Concomitant drugs include all entries in the patient.drug array excluding the suspect ICI agent. Drug categories were identified by string matching: QT-prolonging agents include amiodarone, sotalol, methadone, haloperidol, olanzapine, quetiapine, risperidone, citalopram, escitalopram, venlafaxine, ondansetron, domperidone, azithromycin, clarithromycin, fluconazole, levofloxacin, and ciprofloxacin; beta-blockers include metoprolol, bisoprolol, carvedilol, atenolol, propranolol, nebivolol, and esmolol; diuretics include furosemide, torsemide, bumetanide, hydrochlorothiazide, chlorthalidone, spironolactone, eplerenone, and indapamide. 'Any high-risk' reports the proportion of patients with at least one concomitant agent from any of these three categories. Categories are limited and derived from string matching, so co-reporting is likely to be under-captured.*

**Table S5. Interval from earliest recorded ICI start date to FAERS report receipt, stratified by patient age.**

| **Age stratum** | **n with TTO data** | **Median (days)** | **Q1–Q3 (days)** | **Within first 90 days (%)** |
| --- | --- | --- | --- | --- |
| **<65 yrs** | 596 | 148 | 62–259 | 37.8% |
| **65–74 yrs** | 628 | 99 | 52–215 | 45.9% |
| **≥75 yrs** | 551 | 97 | 53–189 | 47.2% |

*Time-to-onset (TTO) is a proxy metric calculated as the difference in days between the earliest ICI start date (patient.drug.drugstartdate, restricted to ICI agents) and the FAERS event receipt date (receivedate). Reports with missing dates or implausible intervals (≤0 or ≥730 days) were excluded. This is a proxy metric based on the earliest recorded drug-start date and the FAERS receive date; it is not the true clinical onset of myocarditis, which is not captured in FAERS. The interval is shorter in older patients (97–99 days versus 148 days in those aged <65). Because the metric combines true event onset with reporting latency, it is reported as a temporal reporting pattern and not as evidence of earlier clinical onset.*

**Figure S1.** ICI cardiac adverse events by reaction category, with proportion of reports with fatal outcome (FAERS, 2011–2026). Horizontal bars show total report counts pooled across all six ICI agents; bar color encodes the proportion of reports with fatal outcome (red = high, blue = low) per the colorbar legend. Cardiac arrest events show the highest proportion of reports with fatal outcome (78.1%); myocarditis is the largest category.

## **Supplementary Methods S1: openFDA query specification**

FAERS reports were retrieved using the openFDA drug/event endpoint (https://api.fda.gov/drug/event.json). Two retrieval strategies were used. (i) For drug × Preferred Term records (Table 1, Figure 2), separate queries were issued for each combination via search parameter 'patient.drug.openfda.generic_name:DRUG+AND+patient.reaction.reactionmeddrapt:REACTION+AND+receivedate:[20110101+TO+20260331]'. Counts represent records, not unique reports. (ii) For unique-case myocarditis analyses (Tables 2 and 3, Figures 1, 3, and 4), the three myocarditis Preferred Terms were combined within a single OR-query: 'patient.drug.openfda.generic_name:DRUG+AND+(patient.reaction.reactionmeddrapt:"myocarditis"+patient.reaction.reactionmeddrapt:"autoimmune+myocarditis"+patient.reaction.reactionmeddrapt:"immune-mediated+myocarditis")+AND+receivedate:[20110101+TO+20260331]'. The total parameter from the openFDA meta block returns a single de-duplicated case count, with openFDA automatically collapsing multiple submissions sharing the same safetyreportid (FDA's case identifier) to retain only the most recent version, thereby mitigating the well-recognized duplicate-reporting problem in spontaneous pharmacovigilance databases. For the disproportionality analysis, three additional queries per drug obtained: total drug reports across all reactions (a + c), total myocarditis reports across all drugs using the same OR-query (a + b), and the full FAERS background within the date window (a + b + c + d). Calculation followed the standard 2×2 ROR formula. A separate retrieval, run on 26 August 2026 over the same receivedate window, supplied the combination-therapy and concomitant-chemotherapy layer of Supplementary Table S7: for each agent the OR-combined myocarditis query was intersected with a second ICI generic name, and with quoted generic names for anthracyclines, fluoropyrimidines and platinum compounds. Because FAERS adds reports retroactively within a fixed receivedate window, the myocarditis denominators in Supplementary Table S7 run 3–12% above those in Tables 1 to 3, which were retrieved in April 2026; the two retrievals are reported separately and are not cross-tabulated. All reaction and drug names were passed as quoted exact phrases, since unquoted multi-word or hyphenated values tokenise and match substantially more broadly.

**Table S6. Age representation in ICI pivotal trials compared with age distribution of myocarditis reports in FAERS (2011–2026)**

| **Trial** | **Agent(s)** | **Indication** | **N (ITT)** | **% ≥65 (trial)** | **% ≥75 (trial)** | **Reference** |
| --- | --- | --- | --- | --- | --- | --- |
| KEYNOTE-024 | Pembrolizumab | NSCLC 1L (PD-L1 ≥50%) | 305 | 54% | NR | Reck NEJM 2016 |
| KEYNOTE-010 | Pembrolizumab | NSCLC pretreated | 1034 | 43% | NR | Herbst Lancet 2016 |
| KEYNOTE-042 | Pembrolizumab | NSCLC 1L (PD-L1 ≥1%) | 1274 | 45% | 10.1% | Mok Lancet 2019 |
| KEYNOTE-006 | Pembrolizumab | Melanoma 1L | 834 | 33% | NR | Robert NEJM 2015 |
| CheckMate-067 | Nivolumab ± ipilimumab | Melanoma 1L | 945 | 41% | NR | Larkin NEJM 2015 |
| CheckMate-227 | Nivolumab + ipilimumab | NSCLC 1L (PD-L1 ≥1%) | 1189 | 48% | 10% | Hellmann NEJM 2019 |
| CheckMate-214 | Nivolumab + ipilimumab | RCC 1L | 1096 | 38% | 8% | Motzer NEJM 2018 |
| OAK | Atezolizumab | NSCLC pretreated | 850 | 46% | NR | Rittmeyer Lancet 2017 |
| IMpower150 | Atezolizumab + chemo + bev | NSCLC 1L non-squamous | 1202 | 46% | NR | Socinski NEJM 2018 |
| PACIFIC | Durvalumab | Stage III NSCLC post-CRT | 713 | 45% | NR | Antonia NEJM 2017 |
| EMPOWER-CSCC-1 | Cemiplimab | Advanced cutaneous SCC | 193 | Median age 72* | ~34%* | Migden NEJM 2018 |

NR = not reported in age-stratified format. * EMPOWER-CSCC-1 did not report ≥65 as a discrete stratum; the population was predominantly elderly by disease demographics (median 72 years, range 38–95).

**Comparison with FAERS myocarditis reports (this study, aged reports only):**

| **ICI Agent** | **% ≥65 (FAERS)** | **% ≥75 (FAERS)** | **Trial mean ≥65** | **Difference** |
| --- | --- | --- | --- | --- |
| Pembrolizumab | 59.0% | 27.4% | ~43.8% | +15.2 pts |
| Nivolumab | 68.0% | 30.9% | ~42.3% | +25.7 pts |
| Ipilimumab | 66.0% | 26.4% | ~42.3% | +23.7 pts |
| Atezolizumab | 56.4% | 20.4% | ~46.0% | +10.4 pts |
| Durvalumab | 69.9% | 33.2% | ~45.0% | +24.9 pts |
| Cemiplimab | 90.0% | 53.3% | Predominantly elderly | Compatible |

Interpretation: Across the five agents with age-stratified pivotal-trial data, the proportion of unique myocarditis reports in patients aged ≥65 exceeded the corresponding trial representation by 10 to 26 percentage points (pembrolizumab +15.2, nivolumab +25.7, ipilimumab +23.7, atezolizumab +10.4, durvalumab +24.9). The two largest differences, for nivolumab and ipilimumab, should be read alongside Table S7a: 49.5% of nivolumab and 94.1% of ipilimumab myocarditis reports name a second ICI agent, and the trial comparator for both is drawn largely from combination-therapy trials, so neither difference reflects monotherapy exposure. The durvalumab comparison rests on a single trial (PACIFIC). For cemiplimab, EMPOWER-CSCC-1 did not report ≥65 as a discrete stratum, so no difference can be computed; the observed FAERS distribution (90.0% ≥65, 53.3% ≥75) is nonetheless consistent with the elderly demographic of advanced cutaneous squamous cell carcinoma. Because per-drug indication-mix in FAERS differs from any single pivotal trial and FAERS does not provide age-stratified exposure denominators, this comparison is descriptive rather than a formal exposure-adjusted analysis.

**Table S7. Combination therapy and concomitant chemotherapy in unique ICI myocarditis reports (2011–March 2026). Denominators are the unique myocarditis reports for each agent, retrieved by the same OR-combined query used elsewhere in this study but re-run on 26 August 2026; they therefore run slightly above the April 2026 counts in Tables 2 and 3 and should not be cross-tabulated with them**

**Table S7a. ICI–ICI combination therapy in myocarditis reports**

| **ICI Agent** | **Myocarditis reports** | **Co-listing another ICI, n (%)** | **Most frequent partner, n (%)** |
| --- | --- | --- | --- |
| Nivolumab | 1,598 | 791 (49.5%) | Ipilimumab 759 (47.5%) |
| Ipilimumab | 817 | 769 (94.1%) | Nivolumab 759 (92.9%) |
| Pembrolizumab | 2,036 | 105 (5.2%) | Nivolumab 81 (4.0%) |
| Atezolizumab | 480 | 82 (17.1%) | Pembrolizumab 72 (15.0%) |
| Durvalumab | 347 | 60 (17.3%) | Atezolizumab 54 (15.6%) |
| Cemiplimab | 57 | 1 (1.8%) | 1 report naming all six agents |

**Table S7b. Concomitant cardiotoxic chemotherapy co-reporting**

| **ICI Agent** | **Myocarditis reports** | **Any cardiotoxic chemo*, n (%)** | **Platinum, n (%)** | **Anthracycline†, n (%)** | **Fluoropyrimidine, n (%)** |
| --- | --- | --- | --- | --- | --- |
| Pembrolizumab | 2,036 | 571 (28.0%) | 482 (23.7%) | 135 (6.6%) | 46 (2.3%) |
| Nivolumab | 1,598 | 123 (7.7%) | 113 (7.1%) | 7 (0.4%) | 28 (1.8%) |
| Ipilimumab | 817 | 46 (5.6%) | 44 (5.4%) | 2 (0.2%) | 2 (0.2%) |
| Atezolizumab | 480 | 125 (26.0%) | 122 (25.4%) | 20 (4.2%) | 10 (2.1%) |
| Durvalumab | 347 | 80 (23.1%) | 78 (22.5%) | 1 (0.3%) | 2 (0.6%) |
| Cemiplimab | 57 | 5 (8.8%) | 5 (8.8%) | 0 (0.0%) | 0 (0.0%) |

* Includes anthracyclines (doxorubicin, epirubicin, daunorubicin, idarubicin, mitoxantrone), fluoropyrimidines (fluorouracil, capecitabine), and platinum compounds (cisplatin, carboplatin, oxaliplatin). † Anthracyclines represent the chemotherapy class with the strongest established myocarditis/cardiomyopathy signal.

Denominators are unique myocarditis reports retrieved by the OR-combined query of the three myocarditis Preferred Terms, re-run on 26 August 2026 over the same receivedate window (1 January 2011 to 31 March 2026). Because FAERS adds reports retroactively within a fixed window, these counts run 3–12% above the April 2026 retrieval used for Tables 1 to 3; the two retrievals should not be cross-tabulated. Key observations: (1) ICI–ICI co-listing dominates the ipilimumab reports (94.1%) and accounts for about half of the nivolumab reports (49.5%), in both cases almost entirely through the nivolumab–ipilimumab pair (759 reports). It is uncommon for pembrolizumab (5.2%) and cemiplimab (1.8%) but not negligible for atezolizumab (17.1%) or durvalumab (17.3%). (2) Concomitant cardiotoxic chemotherapy is co-reported in a minority of myocarditis reports for every agent (5.6–28.0%), and this co-reporting is overwhelmingly platinum-based (84–100% of the co-reporting for each agent), consistent with routine platinum-containing chemo-immunotherapy rather than with exposure to the classes most strongly linked to myocarditis; anthracycline co-reporting is uncommon throughout (0.0–6.6%). (3) The cemiplimab age skew is not explained by chemotherapy co-exposure: 8.8% of cemiplimab myocarditis reports named any of the three classes and none named an anthracycline. Co-listing indicates that two agents appear in the same case record, not that they were administered concurrently; reports naming two PD-1/PD-L1 agents (for example pembrolizumab and atezolizumab, 72 reports) most plausibly reflect sequential lines of therapy.

**Table S7c. Tyrosine kinase inhibitor co-reporting in unique ICI-associated myocarditis reports**

| **ICI agent** | **Myocarditis reports (n)** | **Set A TKI, n (%)** | **Set B TKI, n (%)** | **Lenvatinib / Cabozantinib / Axitinib (n)** | **Most frequent partner** |
| --- | --- | --- | --- | --- | --- |
| Pembrolizumab | 2,036 | 280 (13.8) | 281 (13.8) | 204 / 4 / 78 | Lenvatinib |
| Nivolumab | 1,598 | 54 (3.4) | 58 (3.6) | 6 / 44 / 7 | Cabozantinib |
| Ipilimumab | 817 | 27 (3.3) | 28 (3.4) | 1 / 23 / 4 | Cabozantinib |
| Atezolizumab | 480 | 8 (1.7) | 13 (2.7) | 4 / 4 / 1 | Lenvatinib / cabozantinib |
| Durvalumab | 347 | 4 (1.2) | 5 (1.4) | 3 / 0 / 1 | Lenvatinib |
| Cemiplimab | 57 | 0 (0.0) | 0 (0.0) | 0 / 0 / 0 | None |

*Set A comprises the tyrosine kinase inhibitors used in approved ICI-containing combination regimens (lenvatinib, cabozantinib, axitinib). Set B adds sunitinib, pazopanib, sorafenib, regorafenib and tivozanib and is not restricted to approved combinations, so it captures sequential as well as concurrent use. Denominators are the unique myocarditis reports per agent retrieved in the same run as the numerators and are therefore internally consistent with Tables S7a and S7b. Co-listing indicates that two agents appear in the same case record; it does not establish concurrent administration. Retrieved September 2026 via the openFDA API using the same quoted generic-name queries, myocarditis Preferred Term set and receivedate window as Tables S7a and S7b.*

**Table S8. Age variable characterization and sensitivity analysis**

**Table S8a. Age availability**

| **Metric** | **N** | **% of records** |
| --- | --- | --- |
| Total cardiac AE drug–reaction records (Table 1) | 18,261 | 100.0% |
| Records with patient.patientonsetage populated | 14,516 | 79.5% |
| Records with age missing | 3,745 | 20.5% |

**Table S8b. Age unit inferred from the recorded value (all six agents)**

| **Recorded value range** | **Inferred unit (E2B code)** | **N** | **% of records with age** |
| --- | --- | --- | --- |
| 3–17 | Decade (800) | 621 | 4.3% |
| 18–110 | Year (801) | 13,812 | 95.2% |
| 111–120 | — | 0 | 0.00% |
| 626–1,028 | Month (802) | 21 | 0.14% |
| 14,233–30,068 | Day (804) | 62 | 0.43% |

Units were inferred from the recorded value, because the value alone identifies the unit unambiguously in this cohort. Values of 3–17 form a distribution peaking at 7–9, matching the age structure of the cohort read in decades (E2B unit 800) rather than a paediatric population; no ICI cardiac adverse event report in this cohort is genuinely paediatric. Values of 626–1,028 correspond to 52–86 years read in months, and values of 14,233–30,068 to 39–82 years read in days. No record falls between 111 and 120. All primary analyses were restricted to ages recorded in years (unit 801).

**Table S8c. Analytic cohort after unit filtering**

| **Metric** | **N** | **% of records with age** |
| --- | --- | --- |
| Excluded: age recorded in decades (unit 800) | 621 | 4.3% |
| Analytic cohort: 18–110 years, recorded in years (unit 801) | 13,812 | 95.2% |
| Excluded: age recorded in months or days (units 802, 804) | 83 | 0.6% |

Sensitivity analysis: age-stratified reporting odds ratios were re-calculated after excluding records with age recorded in units other than years and records missing age. Point estimates changed by less than 1 percent for all six ICI agents; the age-amplification pattern reported in the main manuscript was preserved. Note that decade-coded records are read as ages 3–17 by any query that does not filter on unit, and therefore fall into the '<65' band of data/faers/age_groups_by_drug_reaction.csv in the deposited data; this inflates the record-level '<65' count by 621 (5,407 versus 4,786) and correspondingly deflates the record-level ≥65 proportions cited in Section 3.3. The unique-case myocarditis analyses of Tables 2 and 3 are unaffected, having been retrieved with an explicit age-range filter.
